# Staged management of a giant adult Todani type IVa choledochal cyst mimicking gallbladder hydrops

**DOI:** 10.1093/jscr/rjag833

**Published:** 2026-09-22

**Authors:** Van Trung Hoang, The Huan Hoang, Vichit Chansomphou, Phu Cuong Pham

**Affiliations:** Department of Radiology, Thien Hanh Hospital, 17 Nguyen Chi Thanh Street, Tan An Ward, Dak Lak 63000, Vietnam; Department of Radiology, Thien Hanh Hospital, 17 Nguyen Chi Thanh Street, Tan An Ward, Dak Lak 63000, Vietnam; Department of Radiology, Savannakhet Medical-Diagnostic Center, 266/5 Chaimeuang Phetsarat Street, Kaysone Phomvihane, Savannakhet Province 13000, Lao People's Democratic Republic; Department of General Surgery, Thien Hanh Hospital, 17 Nguyen Chi Thanh Street, Tan An Ward, Dak Lak 63000, Vietnam

**Keywords:** choledochal cyst, bile duct cyst, Todani type IVa, adult, biliary drainage, Roux-en-Y hepaticojejunostomy

## Abstract

Choledochal cysts are uncommon congenital bile duct dilatations, and adult presentation is often complicated by obstruction, cholangitis, pancreatitis, stones, or malignant transformation. We report a 53-year-old woman with obstructive jaundice and severe hepatobiliary injury. Ultrasonography and contrast-enhanced computed tomography showed a giant extrahepatic bile duct cyst measuring ~15–16 cm, marked intrahepatic duct dilatation, gallbladder distension, and enhancing biliary walls without a visible obstructing stone, consistent with a Todani type IVa choledochal cyst complicated by cholangitis. Ultrasound-guided percutaneous biliary drainage was performed, with marked biochemical improvement. Definitive open surgery included cholecystectomy, complete extrahepatic cyst excision, and Roux-en-Y hepaticojejunostomy. This case highlights a surgical pitfall in which a giant bile duct cyst mimics gallbladder hydrops and supports staged decompression before definitive biliary reconstruction when cholangitis is present.

## Introduction

Choledochal cysts are congenital dilatations of the intrahepatic and/or extrahepatic bile ducts. The Todani classification remains the most widely used anatomic framework, and type IVa disease is characterized by combined extrahepatic and intrahepatic bile duct involvement [1, 2]. Although the condition is classically diagnosed during childhood, adult presentation is clinically important because patients often become symptomatic only after complications have developed. Adult choledochal cysts may present with biliary obstruction, recurrent cholangitis, pancreatitis, hepatolithiasis, secondary biliary cirrhosis, or malignancy, and they frequently require multidisciplinary assessment before definitive surgery [3–9].

Giant extrahepatic cystic dilatation in adults is uncommon and may be confused with massive gallbladder hydrops, hepatic cyst, pancreatic pseudocyst, cystic pancreatic neoplasm, or other right upper abdominal cystic lesions. This distinction is not only radiologic but also surgical: isolated gallbladder surgery would be inadequate and potentially hazardous, whereas a choledochal cyst requires careful dissection of the extrahepatic bile duct and biliary-enteric reconstruction. We describe a giant adult Todani type IVa choledochal cyst presenting with obstructive jaundice and suspected cholangitis, managed by staged percutaneous biliary decompression followed by open cyst excision, cholecystectomy, and Roux-en-Y hepaticojejunostomy.

## Case report

A 53-year-old woman was evaluated for obstructive jaundice of unclear cause. Initial biochemical tests demonstrated marked hepatobiliary injury, with total bilirubin 106.3 μmol/L, direct bilirubin 64.4 μmol/L, alanine aminotransferase 792 U/L, aspartate aminotransferase 953 U/L, and gamma-glutamyl transferase 174 U/L. Serum amylase was within the reference range. Upper gastrointestinal endoscopy did not reveal luminal obstruction; the second portion of the duodenum was normal, and only mild antral erythema was reported.

**Figure 1:**
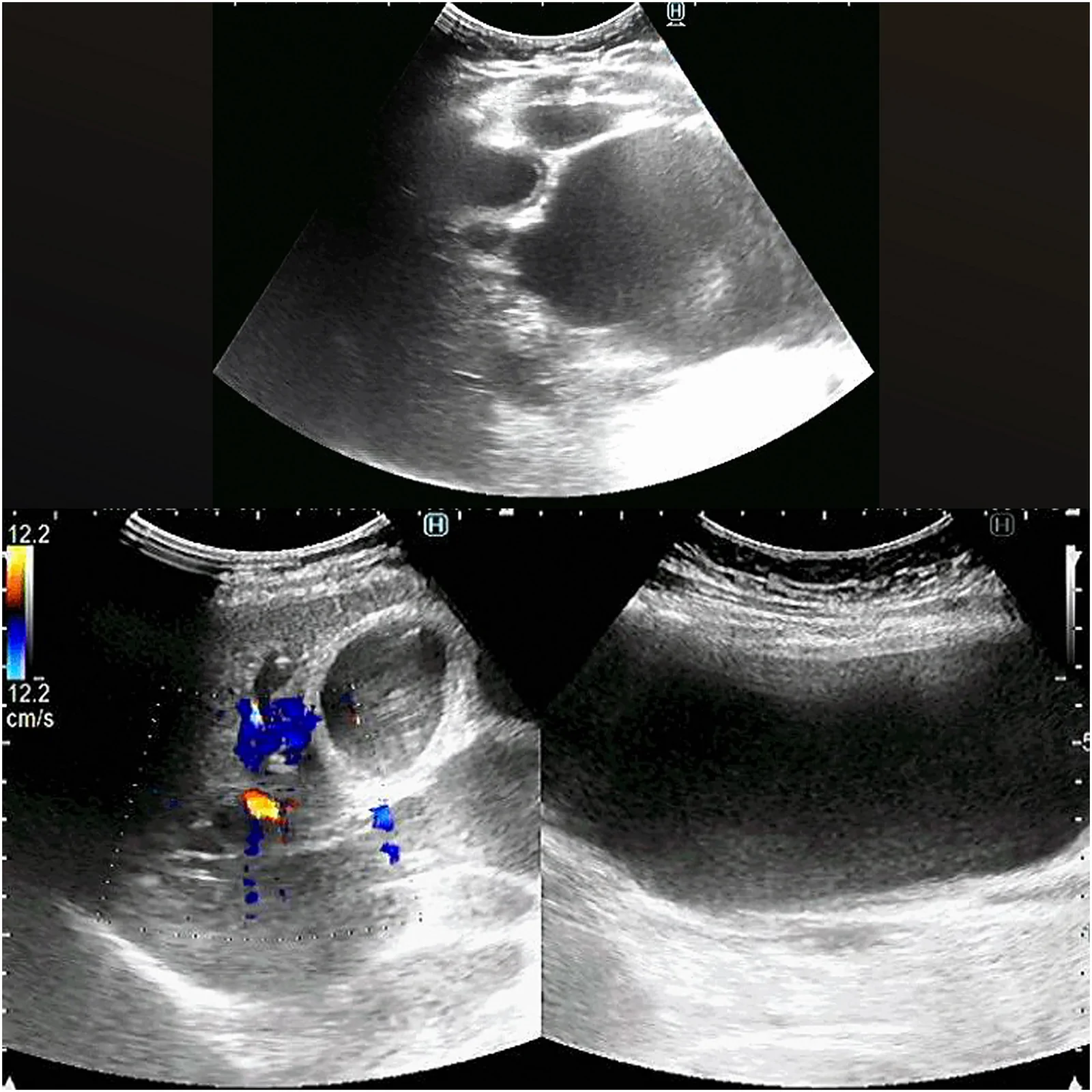
Abdominal ultrasonography showing a giant cystic extrahepatic bile duct in the subhepatic region, associated intrahepatic biliary dilatation, and gallbladder distension.

Abdominal ultrasonography demonstrated marked intrahepatic bile duct dilatation without stones, a markedly dilated cystic extrahepatic bile duct measuring ~164 × 71 mm, and a distended gallbladder measuring ~97 × 83 mm (Fig. 1). Because the patient had severe biliary obstruction with suspected cholangitis, ultrasound-guided percutaneous biliary drainage was performed for decompression. Bile culture was negative. Liver tests improved after drainage: alanine aminotransferase decreased to 302 U/L and then 64 U/L, aspartate aminotransferase decreased to 136 U/L and then 39 U/L, and total bilirubin decreased to 27.3 μmol/L and then 13.9 μmol/L.

**Figure 2:**
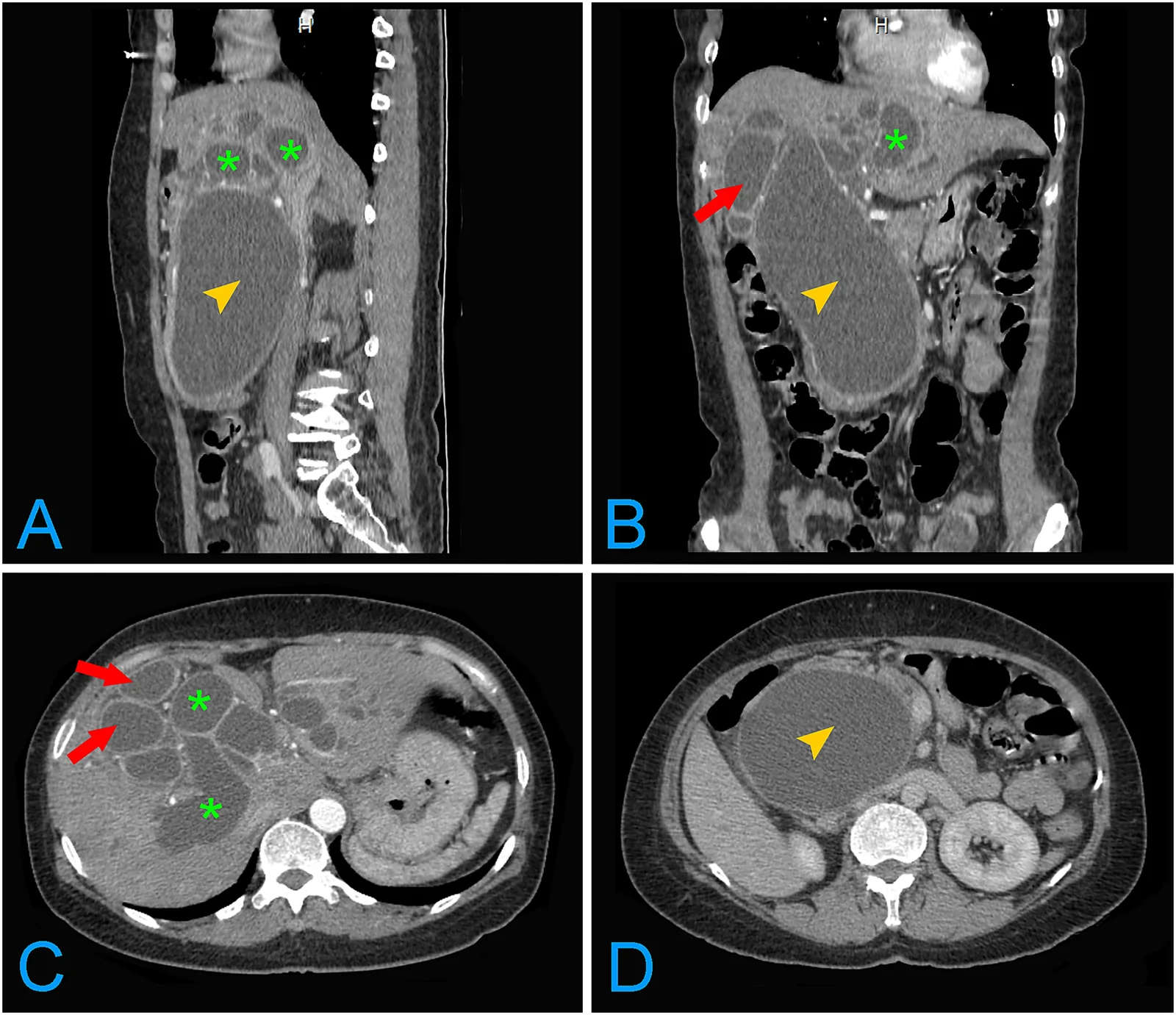
Contrast-enhanced CT. Sagittal (A), coronal (B), and axial (C, D) images demonstrate a giant cystic dilatation of the extrahepatic bile duct (arrowheads), marked intrahepatic biliary dilatation (asterisks), and a distended gallbladder (arrows), compatible with Todani type IVa choledochal cyst.

Multiphasic abdominal computed tomography (CT), including non-contrast and contrast-enhanced phases, showed a giant cystic dilatation of the common bile duct measuring ~80 × 100 × 150 mm, with marked intrahepatic duct dilatation (Fig. 2). The biliary walls enhanced after contrast administration, and heterogeneous hepatic perfusion changes were present, supporting inflammatory obstruction or cholangitis. The gallbladder was distended but separate from the giant cystic bile duct. No radiopaque biliary stone, pancreatic mass, pancreatic duct dilatation, or obstructing distal common bile duct stone was identified. These findings supported a diagnosis of Todani type IVa choledochal cyst rather than isolated gallbladder hydrops.

**Figure 3:**
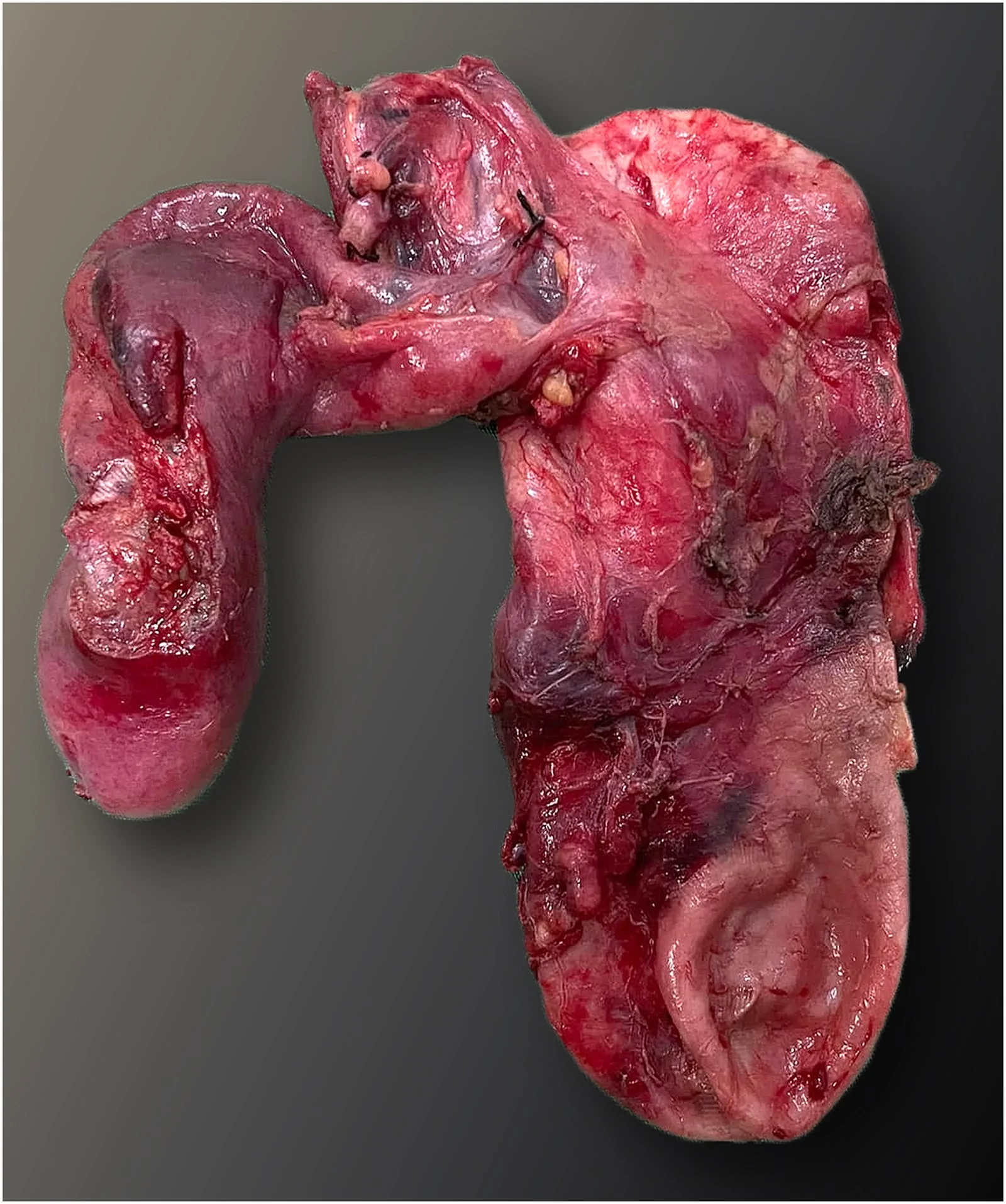
Gross surgical specimen after cholecystectomy and extrahepatic bile duct cyst excision, demonstrating the markedly dilated cystic bile duct segment and attached gallbladder.

After decompression and clinical stabilization, definitive open surgery was performed. At exploration, a large common bile duct cyst measuring ~70 × 100 × 100 mm occupied the subhepatic space, extended posterior to the duodenum, and partially surrounded the pancreatic head. The gallbladder was not tense, confirming that the dominant cystic lesion was the bile duct cyst rather than gallbladder hydrops. The surgical team performed cholecystectomy, complete excision of the extrahepatic cystic bile duct up to the level of the common hepatic duct, and Roux-en-Y hepaticojejunostomy (Fig. 3). Subhepatic and peripancreatic drains were placed, and the specimen was submitted for histopathologic evaluation. No immediate operative complication was documented in the available medical record.

## Discussion

This case demonstrates several practical points in the management of adult choledochal cysts. First, adult disease should not be regarded as an incidental congenital anomaly. In adult series, choledochal cysts are often diagnosed after biliary obstruction, cholangitis, stone disease, pancreatitis, or malignancy has already occurred [5–9]. In our patient, the severe bilirubin elevation, marked transaminase abnormality, duct-wall hyperenhancement, and transient hepatic perfusion abnormality were consistent with inflammatory biliary obstruction. A negative bile culture does not exclude cholangitis, particularly when culture timing, drainage, and antimicrobial exposure may affect yield.

Second, the preoperative distinction between a giant bile duct cyst and gallbladder hydrops is critical. A giant extrahepatic bile duct cyst may occupy the subhepatic region and resemble a markedly distended gallbladder on initial imaging. Helpful features include continuity with the biliary tree, disproportionate extrahepatic bile duct dilatation, associated intrahepatic duct dilatation, and identification of a separate gallbladder. Cross-sectional imaging is also important for assessing the relationship of the cyst to the pancreatic head, duodenum, portal vein, and hepatic hilum. In this case, the operative finding of a non-tense gallbladder was particularly useful because it confirmed the imaging impression that the dominant cystic structure was not the gallbladder itself. Magnetic resonance cholangiopancreatography (MRCP) is often the most comprehensive non-invasive method for biliary mapping, but ultrasonography and CT can define the major anatomy and identify complications when MRCP is unavailable [10, 11].

Third, staged management may be useful when a giant choledochal cyst is complicated by obstruction or cholangitis. In the present case, percutaneous biliary drainage was used as a bridge to definitive surgery and was followed by rapid biochemical improvement. This approach can reduce intraductal pressure and inflammatory burden before a technically demanding biliary reconstruction, particularly when the cyst is large, infected, or closely related to the duodenum and pancreatic head. It also allows time to correct dehydration, electrolyte abnormalities, malnutrition, or coagulopathy before a long hepatobiliary operation. Drainage alone, however, is not definitive treatment because it does not remove the abnormal ductal epithelium and does not eliminate the long-term risks of recurrent cholangitis, stones, anastomotic problems, or malignancy.

Definitive treatment for type I and type IV choledochal cysts generally consists of complete extrahepatic cyst excision with cholecystectomy and Roux-en-Y hepaticojejunostomy [12, 13]. Internal drainage procedures have largely been abandoned because of recurrent infection and persistent malignancy risk. A meta-analysis reported malignant transformation in ~11% of choledochal malformations, with risk concentrated in type I and type IV disease, and risk after drainage procedures higher than after complete excision [14]. Operative principles include safe proximal transection near the common hepatic duct, distal dissection as low as feasible without pancreatic duct injury, and a tension-free biliary-enteric anastomosis. Even after apparently complete resection, long-term surveillance is recommended because biliary malignancy can arise from residual intrahepatic ducts, the hepatic hilum, anastomotic region, or intrapancreatic remnant [15]. In type IVa disease, residual intrahepatic duct dilatation may persist after extrahepatic cyst excision; follow-up should therefore assess liver tests, intrahepatic stones, recurrent cholangitis, anastomotic stricture, and biliary neoplasia.

This report is limited by the absence of MRCP, detailed histopathology, and long-term postoperative follow-up data. Nevertheless, the concordant ultrasonographic, CT, and operative findings provide strong anatomic correlation and illustrate a safe management sequence for a giant adult type IVa choledochal cyst. The case is also relevant for non-tertiary settings where MRCP may not be immediately available but timely decompression and surgical referral remain essential. In conclusion, when a large right upper abdominal cystic lesion is associated with intrahepatic biliary dilatation, a choledochal cyst should be actively considered. Temporary decompression can be a useful bridge in patients with obstruction or cholangitis, but complete cyst excision and biliary-enteric reconstruction remain the central curative steps.
